# Executive functions and self‐limited epilepsy with centro‐temporal spikes: A scoping review

**DOI:** 10.1002/epd2.70176

**Published:** 2026-01-12

**Authors:** Edoardo Fino, Martina Calì, Sara Senese, Simona Pellacani, Viola Margheri, Chiara Pecini, Carmen Barba

**Affiliations:** ^1^ Neuroscience, Pharmacology and Child Health Department University of Florence Florence Italy; ^2^ Neuroscience and Human Genetics Department, Meyer Children's Hospital IRCCS Florence Italy; ^3^ Department of Education, Languages, Intercultures, Literatures and Psychology University of Florence Florence Italy

**Keywords:** executive functions, self‐limited epilepsy with centro‐temporal spikes

## Abstract

Executive functions are a set of high‐level cognitive processes necessary for planning, organization, decision‐making, self‐control, and attention, and are carried out in the anterior frontal lobes. An impairment in executive functioning might present as difficulties in planning and organizing activities, in attention and concentration, in cognitive flexibility, impulsiveness, and working memory fragility. These might result in greater emotional and psychopathological difficulties and poorer academic performance. Self‐limited epilepsy with centro‐temporal spikes (SeLECTS), the most common epileptic syndrome occurring in the pediatric population, is characterized by seizure remission around puberty in most cases. However, despite the favorable seizure outcome, previous studies have suggested that executive function deficits might be present and persist after epilepsy remission. We conducted a scoping review to investigate the current knowledge on executive functioning in children with SeLECTS. Furthermore, we explored psychopathological and emotional dimensions and daily functioning in this population. Starting from two reviews published in 2021, we conducted a complementary search and included 41 articles, from which we analyzed clinical data, neuropsychological findings, and their respective correlations. Our results confirmed the possible presence of executive dysfunction in patients with SeLECTS in the domains of inhibition and cognitive flexibility. We also strengthen possible impairments in working memory and higher order executive functions. We confirmed the correlation between executive dysfunction and both early age at onset and high frequency of electroencephalogram abnormalities and observed a possible role for high seizure frequency, secondary bilateralization, and the use of anti‐seizure medications. We also found a higher prevalence of psychopathological dimensions, most commonly attention deficit—hyperactivity disorder, compared with controls. Overall, our findings support the need for neuropsychological assessment in clinical practice for children with SeLECTS to characterize executive functioning and its impact on psychopathological and emotional dimensions, as well as academic performance.

**Key points**

- We conducted a scoping review to investigate the current knowledge on executive functioning in children with SeLECTS.
- We confirm the presence of executive dysfunction in patients with SeLECTS in the domains of inhibition and cognitive flexibility.
- Executive dysfunction correlates with early age at onset and high frequency of electroencephalogram abnormalities in children with SeLECTS.
- The prevalence of attention deficit – hyperactivity disorder is higher in children with SeLECtS compared to controls.

## 1 INTRODUCTION

Self‐limited epilepsy with centro‐temporal spikes (SeLECTS), formerly named “benign rolandic epilepsy” (BRE) or “benign epilepsy with centro‐temporal spikes” (BECTs), is the most common epileptic syndrome occurring in the pediatric population.^1^ It typically presents in children with normal psychomotor development and normal brain MRI, between 3 and 14 years, with rare and brief seizures predominantly occurring during sleep, affecting the orofacial and brachial regions. The EEG typically shows high‐amplitude bi‐ or tri‐phasic spike complexes in the centro‐temporal regions in normal background activity, activated during drowsiness and sleep. Seizures usually remit by puberty.^2^

Despite a favorable seizure prognosis, SeLECTS has been associated with a higher prevalence of psychopathological dimensions than in the general pediatric population—particularly inattentive‐type ADHD,^3^ as well as unfavorable long‐term academic and social outcomes extending into adulthood,^4^, ^5^ and evidence of executive dysfunctions.^6^, ^7^

Executive functions (EFs) refer to a set of top‐down mental processes required in situations that demand focus and attention, in which relying on automatic responses, instinct, or intuition would be inadequate or inappropriate. Executive functions are categorized into basic EFs, which include working memory, inhibition, and cognitive flexibility, and higher order EFs, which encompass planning, problem‐solving, and metacognition.^8^ EFs are considered essential for cognitive, social, and psychological development, as well as for overall mental and physical health.^9^, ^10^ An impairment in EFs development is recognized as a transdiagnostic feature across pediatric neurodevelopmental disorders, with severity increasing alongside diagnostic complexity.^11^

Therefore, the integrity of EFs can be used as a predictor of school performance and quality of life, in patients with epilepsy.^12^

Difficulties in cognitive flexibility, particularly in shifting strategies during problem‐solving, as well as planning and organization tasks have been previously described in children with SeLECTS as well as in verbal fluency, reading comprehension, processing speed, and inhibition.^6^, ^7^

However, studies on EF dysfunction in SeLECTS are heterogeneous with respect to study design, sample characteristics, and assessment instruments, thereby precluding drawing definite conclusions. In addition, despite the availability of several diagnostic tools specifically developed to assess EFs,^13^ there is a lack of standardization in the assessment procedures applied to individuals with SeLECTS, as well as an absence of specific recommendations for their long‐term longitudinal monitoring.

Therefore, the aim of our scoping review was to analyze pooled literature data from selected studies to investigate existing knowledge on the frequency of executive dysfunctions, psychopathological and emotional dimensions, and daily functioning alterations in patients with SeLECTS, as well as the factors influencing these deficits, with particular attention to clinical and methodological variables.

## 2 METHODS

We conducted this scoping review in accordance with the checklist outlined by Tricco and colleagues.^14^ This scoping review was preregistered on the Open Science Framework (OSF; DOI: https://doi.org/10.17605/OSF.IO/AZ9HP). Our research question, defined by the Population–Concept–Context (PCC) question development framework, was related to possible EF dysfunction, globally or within specific domains (i.e., working memory, inhibition, cognitive flexibility, planning, or problem‐solving), and any eventually associated learning disabilities in children with SeLECTS without specifying a particular context or setting.^2^, ^15^ We preliminarily assessed the breadth of our research question by verifying whether systematic or scoping reviews on the topic had already been conducted, and we ensured that sufficient literature was available to justify our work.

Having identified two recent systematic reviews on topics relevant to our research question, we initially selected all the publications they included.^6^, ^7^

Subsequently, we conducted a search across three medical and scientific literature databases (Pubmed, Web of Science, and Embase), restricting the quest to studies published from 2021 onward. We used the syntax terms “Rolandic Epilepsy,” “Benign Epilepsy With Centrotemporal Spikes,” “Temporal‐Central Focal Epilepsy,” “Self‐limited Epilepsy with Centrotemporal Spikes,” “SeLECTS,” “BECTS,” “BCETCS,” “BRE,” and “BECRS” to address SeLECTS, and “Cognition,” “Neuropsychology,” “Neuropsychological Tests,” “Executive Functions,” “Executive Control,” “Working Memory,” “Inhibition,” “Cognitive Flexibility,” “Shifting,” “Planning,” and “Problem Solving.” We only included articles involving human subjects, published in peer‐reviewed journals, and in English language. We excluded abstracts, posters, editorial letters, and narrative reviews.

After removing duplicates and conducting content analysis, we screened articles by title and abstract and then selected the final collection of articles by full‐text analysis. We set inclusion criteria as follows: (1) diagnosis of SeLECTS; (2) age < 18 years old; (3) assessment of EFs either in general or in specific domains, that is, working memory, inhibition, cognitive flexibility, planning, and problem‐solving, using standardized neuropsychological tools. We excluded studies enrolling patients with other self‐limited or structural epilepsies if we could not extrapolate data on patients with SeLECTS.

We collected data extracted from all the selected articles in a dedicated dataset, including clinical data (number of patients with SeLECTS, gender, age at the evaluation, age at seizure onset, epilepsy duration, type and frequency of seizures, type of anti‐seizure medication [ASM], if any, and sleep disorder, if any), EEG data (lateralization of interictal epileptiform abnormalities, that is, left, right, or bilateral, quantitative analysis of interictal activity), type of neuropsychological and in particular EFs assessment (type and results of tests, and setting of evaluation). Given the close relationship between EFs and other psychological domains, we also extracted—from both the included screened studies—the results of any assessments targeting psychopathological dimensions, including internalizing and externalizing problems, affective and mood‐related traits, as well as dimensions linked to anxiety, somatic complaints, emotional difficulties, and their associations with executive functioning. In addition, we evaluated whether the authors complemented the clinical assessment with patient‐ or caregiver‐reported questionnaires to further characterize patients' daily functioning from their own perspective.

The statistical approaches reported in the included studies (such as correlation analyses, regression models, and group comparisons) were analyzed when mapping the associations between clinical characteristics and EFs performance, psychopathological and emotional dimensions, and daily functioning. Results were summarized descriptively, with attention to the type of statistical test employed and the significance of the reported associations.

Lastly, based on the results obtained from the present scoping review and expert opinions on this topic, we have developed a proposal for a standardized protocol to assess EFs and explore psychopathological and emotional dimensions and daily functioning in patients with SeLECTS.

## 3 RESULTS

### 3.1 Study selection

**FIGURE 1:**
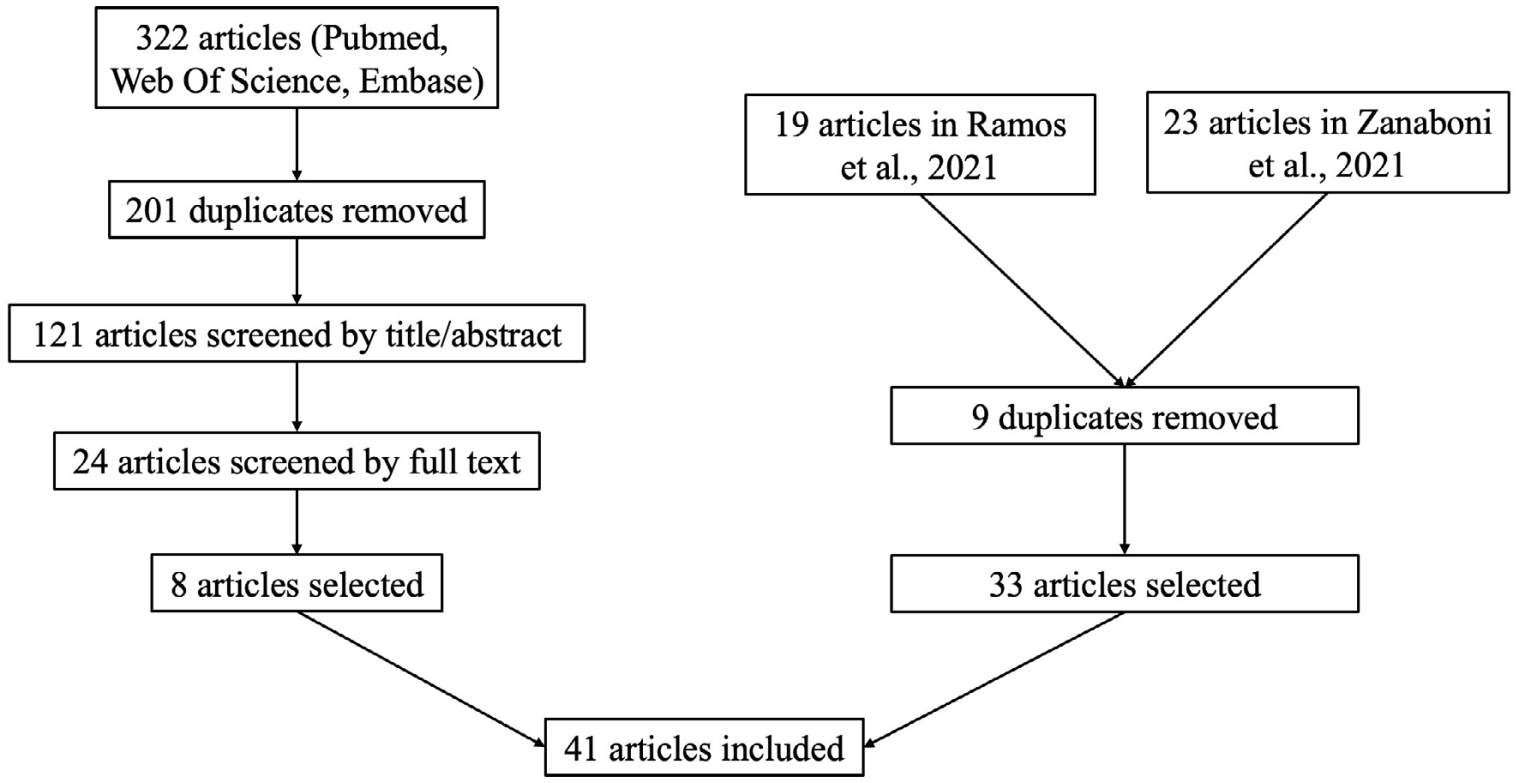
Flowchart of the study selection.

We initially included 19 studies from the meta‐analysis by Ramos et al. (2022, Epub 2021) and 23 articles from the systematic review by Zanaboni et al. (2021), as total of 33 papers after removing nine duplicates.^6^, ^7^ Then, as illustrated in Figure 1, our search for studies published after 2021 through Pubmed, Web of Science and Embase yielded 322 studies. After removing 201 duplicates, we identified 121 unique studies, of which we selected 24 based on their title and abstract. Following full‐text analysis, eight papers met the inclusion criteria for this scoping review, bringing the final number of studies included for the analysis to 41.

### 3.2 Demographical and clinical information

The selected 41 papers collectively provided data on 1191 patients diagnosed with SeLECTS (Table 1). Gender data were available for 1142 patients from 39 studies, of whom 528 were females (46.2%). The mean age at the time of the assessment was reported for 1178 patients from 40 studies and corresponded to 9.8 years (±1.4, range 5.8–12.5). Age at seizure onset was available for 1023 patients (35 studies), being on average 7.2 years (±1.3, range 4.9–9.9), whereas the mean duration of active epilepsy, reported for 1010 patients across 34 articles, was 1.9 years (±1.8 years, range 0.0–7.0).

**TABLE 1:** Features of the 41 selected studies.

Authors	Year	Study design	Sample size	Gender		Mean Age	Mean age at onset	Mean epilepsy duration	EEG lateralization			No of patients treated with ASM
				M	F	Years	Years	Years	Left	Right	Bilateral	
Croona et al.^16^	1999	Case–control	17	7	10	12.5	5.5	7	–	–	–	12
Lindgren et al.^17^	2004	Case–control	26	13	13	12.7	7.1	5.6	–	–	–	13
Duman et al.^18^	2008	Case–control	21	11	10	8.9	6.2	2.7	9	7	2	0
Danielsson et al.^19^	2009	Case–control	25	11	14	5.1	4.3	0.8	5	7	1	10
Miziara et al.^20^	2012	Case–control	40	15	8	8	6.7	1.3	10	11	12	22
Neri et al.^21^	2012	Case–control	25	14	11	10.9	5.7	5.2	7	7	11	17
Garcia‐Ramos et al.^22^	2015	Case–control	24	13	11	10.5	9.8	0.7	–	–	–	15
Malfait et al.^23^	2015	Case–control	15	12	3	11.1	7.8	3.3	6	5	4	12
Xiao et al.^24^	2015	Case–control	73	41	32	9.7	8.9	0.8	30	33	10	30
Yang et al.^25^	2015	Case–control	90	57	33	8.5	7.1	0.4	32	34	21	0
Filippini et al.^26^	2016	Case–control	15	9	6	9.2	9.2	0	2	5	8	0
Cheng et al.^27^	2017	Case–control	47	19	28	9.6	–	–	–	–	–	44
Lima et al.^28^	2017	Case–control	20	7	13	10.9	6.6	4.3	–	–	–	14
Elkholy et al.^29^	2018	Case–control	30	17	13	8.8	6.9	1.9	15	15	0	22
Kagitani‐Shimono et al.^30^	2018	Case–control	10	3	7	10.7	5.8	4.9	1	1	8	7
Kim et al.^31^	2014	Case–control	19	11	8	10.7	7.4	3.3	6	4	9	14
Lima et al.^32^	2018	Case–control	23	15	8	11.2	7	3	7	4	4	17
Ay et al.^33^	2009	Case–control	35	19	16	10.4	–	–	15	11	9	18
Lin et al.^34^	2012	Case–control	13	8	5	10.2	9.5	0.5	–	–	–	8
Garcia‐Ramos et al.^35^	2019	Case–control	19	13	6	10.5	9.9	0.6	–	–	–	11
Vintan et al.^36^	2012	Case–control	18	13	5	8.9	7	1.9	13	5	0	0
Filippini et al.^37^	2015	Case–control	23	15	8	8.8	6.5	2.3	–	–	–	–
Piccinelli et al.^38^	2008	Case–control	20	8	12	10.3	7.8	2.5	–	–	–	16
Ayaz et al.^39^	2013	Case–control	31	18	13	10.2	8.1	2.1	10	12	9	24
Banaskiwitz et al.^40^	2017	Case–control	30	18	12	10.5	–	–	10	9	8	13
Cerminara et al.^41^	2010	Case–control	21	12	9	9.9	9.8	0.1	–	–	10	0
Ciumas et al.^42^	2020	Case–control	17	12	5	9.7	7.2	2.4	8	7	2	8
Ciumas et al.^43^	2014	Case–control	25	18	7	9.6	7.7	1.8	14	2	9	10
Datta et al.^44^	2013	Case–control	27	14	13	9.9	7.9	2	8	15	4	15
Teixeira et al.^45^	2020	Case–control	30	18	12	9.9	6.8	3.2	7	12	10	13
Verrotti et al.^46^	2013	Case–control	9	5	4	7.8	–	–	4	2	3	0
Leoncio et al.^47^	2021	Case–control	21	12	9	9.1	–	–	–	–	–	–
Goldberg et al.^48^	2009	Case–control	36	–	–	9.5	5.8	3.7	–	–	–	0
Sreenivasan et al.^49^	2022	Case–control	22	15	7	10.6	8	2.6	8	4	10	20
Zanaboni et al.^50^	2024	Multicenter observational	129	41	88	11.5	6.8	3.1	–	–	–	67
Sousa et al.^51^	2023	Case–control	18	11	7	8.7	7.3	1.5	9	5	4	8
Wu et al.^52^	2021	Case–control	42	24	18	8.5	8.4	0.1	18	16	8	40
Shi et al.^53^	2025	Case–control	36	17	19	11.6	7.6	4.1	–	–	–	34
Duma et al.^18^	2021	Case–control	13	–	–	–	6.3	–	0	5	7	2
Ragab et al.^54^	2024	Case–control	22	12	10	8.4	7.5	0.9	–	–	–	0
Chen et al.^55^	2025	Case–control	26	12	14	9.5	–	–	–	–	–	–

Abbreviations: ASM, anti‐seizure medications; F, female; M, male.

Authors described seizure frequency in two ways: either as the mean number of lifetime seizures, in eight studies involving 329 patients, with an average of 5.8 episodes since onset; or using frequency categories, in seven studies including 261 patients: among these, 141 patients (54.0%) experienced yearly seizures, 19 (7.3%) monthly seizures, three (1.1%) sporadic seizures, and two (0.8%) weekly seizures. In addition, six patients (2.3%) had seizures of unknown frequency, and 89 (34.1%) were seizure‐free.

Eleven studies reported the number of patients who had experienced seizures with focal to bilateral generalization, amounting to 183/392 patients (46.7%). Only five studies reported the number of patients experiencing seizures during daytime wakefulness, totalizing 21 patients out of 108 (19.4%).

We extracted information regarding pharmacological treatment on 1121 patients from 38 studies, of which 552 (49.2%) were treated with ASM (anti‐seizure medications). Among the 24 studies that specified the number of ASM used, 364/412 patients (88.3%) were on monotherapy and 48/412 patients (11.7%) on polytherapy. With respect to the type of ASM, frequency of administration was reported for 266 patients from 16 studies: the most frequently used drug was valproate (91 patients, 34.2%), followed by oxcarbazepine (58 patients, 21.8%), carbamazepine (34 patients, 12.8%), levetiracetam (27 patients, 10.2%), sulthiame (25 patients, 9.4%), clobazam (nine patients, 3.4%), lamotrigine (eight patients, 3.0%), phenytoin (two patients, 0.8%), topiramate (two patients, 0.8%), and zonisamide (one patient, 0.4%).

We found information about EEG characteristics in 25 out of 41 studies, including 691 patients. Specifically, 254 patients (36.8%) showed left‐sided lateralization of epileptiform abnormalities, 238 patients (34.4%) right‐sided lateralization, and 173 patients (25.0%) bilateral abnormalities. The remaining 26 patients (3.8%) showed no abnormalities.

In nine studies and 291 patients, authors conducted a quantitative analysis of EEG abnormalities, with great heterogeneity in terms of methodology. In two studies comprising 32 patients, the mean frequency of abnormalities per minute throughout the entire recording was 19.5.^30^, ^49^ In one study by Cerminara et al.^41^ including 21 patients, the frequency of epileptiform abnormalities exceeded 10 per minute in 11 patients, was below 5 per minute in nine patients, and could not be classified in one patient. Filippini et al.^37^ recorded a mean of 27.4 abnormalities per minute during non‐REM sleep on 23 patients. Vintan et al.^36^ provided separated wakefulness and sleep EEG data of 18 patients and documented on average 11.2 and 27.0 abnormalities per minute, respectively. In a study by Miziara et al.^20^ including 40 patients, 17 (42.5%) displayed more than 10 abnormalities in 5 min of recording, and 23 (57.5%) <10. Yang et al.^25^ recorded, in a cohort of 90 patients, a mean of 5.2 abnormalities during wakefulness and 28.9 abnormalities during sleep, calculated in the most active minute. Elkholy et al.^29^ reported a mean of 57% of 10‐second EEG epochs containing abnormalities relative to the total number of 10‐second epochs in the EEG recordings on 30 patients. Finally, Datta et al.^44^ mentioned “high” values of epileptic abnormalities in three of the 27 enrolled patients, without specifying the measurement criteria.

In addition, we collected information regarding sleep quality on 43 patients from two studies. Filippini et al.^37^ described two patients (4.7%) presented with non‐REM parasomnias of the sleep terrors (pavor nocturnus) type, while Ragab et al.^54^ reported alterations in total sleep time, sleep latency, REM latency, wake after sleep onset, sleep efficiency, sleep fragmentation, sleep stage transition index, arousal index, periodic limb movement index, and the percentages of N2, N3, and REM sleep relative to total sleep time, as well as the REM sleep without atonia (RSWA) index in 22 patients (51.2%) who underwent polysomnography, compared with healthy controls.

In 39 out of 41 studies, a total of 1.109 healthy controls were evaluated, predominantly matched for age and sex, of which eight from one study were healthy siblings of the enrolled patients, serving as a third comparison group alongside healthy controls.^46^

### 3.3 Neuropsychological evaluation

With respect to setting, in all studies patients physically attended hospital facilities to complete neuropsychological evaluations. In seven studies, 265 patients were evaluated using a computer‐based test, which was still carried out on‐site.

Cognitive abilities were assessed using the Wechsler intelligence scales in 698 patients, with significantly lower results in 160 (22.9%) when compared with healthy controls. In addition, the Progressive Matrices of Raven were administered in 130 patients, none of which showed pathological results. Table 2 summarizes all the neuropsychological tests used in each study, classified according to the primary executive function investigated, that is, basic EFs (working memory, inhibition, and cognitive flexibility) or higher order EFs (planning/problem‐solving).^8^

**TABLE 2:** Assessment tests and scoring of executive functions.

Study	Working memory			Inhibition			Cognitive flexibility			Planning/problem‐solving		
	Test	Sample size	Deficit	Test	Sample size	Deficit	Test	Sample size	Deficit	Test	Sample size	Deficit
Croona et al.^16^	Digit Span	17	N	Phonological verbal fluency	17	Y	Trail‐making test	17	N	Complex Figure of Rey	17	N
	Block Span	17	N	Tower of London	17	Y	Phonological verbal fluency	17	Y	Tower of London	17	Y
	RAVLT	17	Y				Tower of London	17	Y			
	Story recall	17	Y									
	Spatial learning test	17	N									
	Phonological verbal fluency	17	Y									
Lindgren et al.^17^	Digit Span	26	N				Trail‐making test	26	Y	Complex Figure of Rey	26	N
	Block Span	26	N	Tower of London	26	Y	Tower of London	26	Y	Tower of London	26	Y
	RAVLT	26	Y									
	Story recall	26	Y									
	Spatial learning test	26	N									
	Phonological verbal fluency	26	Y	Phonological verbal fluency	26	Y	Phonological verbal fluency	26	Y			
Duman et al.^56^							Wisconsin Card Sorting Test	21	N			
Danielsson et al.^19^				KET‐KID	25	Y	KET‐KID	25	Y			
Miziara et al.^20^							Trail‐making test	31	N			
Neri et al.^21^	Phonological verbal fluency	25	Y	Phonological verbal fluency	25	Y	Phonological verbal fluency	25	Y			
	WRAML	25	N				Trail‐making test	25	Y			
							Wisconsin Card Sorting Test	25	Y			
Garcia‐Ramos et al.^22^	Children's Memory Scale	24	Y	Digit Symbol—Coding	24	Y						
Malfait et al.^23^				Everyday Attention for Children test	15	Y						
				D‐KEFS	15	Y						
Xiao et al.^24^	Phonological verbal fluency	73	Y	Phonological verbal fluency	73	Y	Phonological verbal fluency	73	Y			
							Trail‐making test	73	Y			
Yang et al.^25^				Attention network test	90	Y						
Filippini et al.^26^	Digit span	15	N	Five‐point test	15	Y	Five‐point test	15	Y	Five‐point test	15	Y
	Alpha span test	14	N									
Cheng et al.^27^							Wisconsin Card Sorting Test	47	Y			
Lima et al.^28^	Block span	20	N	MFFT	20	Y	Wisconsin Card Sorting Test	20	Y			
	WRAML	20	N				Trail‐making test	20	Y			
Elkholy et al.^29^	Digit Span	30	Y	Digit Symbol	30	Y	Trail‐making test	30	N	Block design test	30	Y
	Spatial memory test	30	Y	Letter cancellation test	30	Y						
Kagitani‐Shimono et al.^30^	BRIEF	10	Y	BRIEF	10	Y	BRIEF	10	Y	BRIEF	10	Y
				Stroop Test	10	N						
Kim et al.^31^	RAVLT	19	N	Phonological verbal fluency	19	N	Phonological verbal fluency	19	N	Complex figure of Rey	19	N
	Phonological verbal fluency	19	N									
Lima et al.^32^	Digit span	23	Y				Trail‐making test	23	Y			
	Phonological verbal fluency	23	N	Phonological verbal fluency	23	N	Phonological verbal fluency	23	N			
	WRAML	23	N				Wisconsin Card Sorting Test	23	Y			
	Similarities	23	N	Similarities	23	N	Similarities	23	N			
	Picture Concepts	23	N	Picture Concepts	23	N	Picture Concepts	23	N			
	Letter‐Number sequencing	23	N									
Ay et al.^33^	Digit Span	32	N	Stroop test	32	N						
Lin et al.^34^	BRIEF	13	_	D‐KEFS	13	_	BRIEF	13	_	BRIEF	13	_
	D‐KEFS	13	_									
Garcia‐Ramos et al.^35^	Children's memory scale	19	N	Digit Symbol	19	N						
				D‐KEFS	19	N						
Vintan et al.^36^	CANTAB	18	N									
Filippini et al.^37^	Phonological verbal fluency	23	N	Phonological verbal fluency	23	N	Phonological verbal fluency	23	N			
	Test of Memory and Language	23	N									
Piccinelli et al.^38^	Digit Span	20	N	Conners continuous performance test	20	N						
	TOMAL	20	N	TOMAL	20	N						
Ayaz et al.^39^				Stroop test	31	Y	Wisconsin Card Sorting Test	31	N			
Banaskiwitz et al.^40^				Stroop test	30	Y	COWAT	30	Y	COWAT	30	Y
				Tower of London	30	N	Modified Card Sorting Test	30	Y	Tower of London	30	N
							Tower of London	30	N			
Cerminara et al.^41^				Go/No‐Go Task	21	Y						
Ciumas et al.^42^	Digit Span	17	Y									
	Sternberg maintenance task	17	Y									
Ciumas et al.^43^	Digit Span	25	Y									
	Letter‐Number sequencing	25	Y									
Datta et al.^44^	Phonological Verbal Fluency	27	N	Phonological verbal fluency	27	N	Phonological verbal fluency	27	N			
	Corsi block tapping test	27	N	Semantic verbal fluency	27	N						
Teixeira et al.^45^	Phonological Verbal Fluency	30	Y	Phonological verbal fluency	30	Y	Phonological verbal fluency	30	Y			
	Verbal memory screening test	30	N	Semantic verbal fluency	30	Y						
Verrotti et al.^46^	NEPSY‐II	9	Y	NEPSY‐II	9	Y	NEPSY‐II	9	Y	NEPSY‐II	9	Y
Leoncio et al.^47^	Digit Span	21	Y									
	Block Span	21	Y									
	Phonological Verbal Fluency	21	Y	Phonological verbal fluency	21	Y	Phonological verbal fluency	21	Y			
	Corsi block tapping test	21	Y									
Goldberg et al.^48^	Digit Span	36	Y	Semantic verbal fluency	36	Y	Phonological verbal fluency	36	Y	Complex figure of Rey	36	N
	RAVLT	36	N	Phonological verbal fluency	36	Y	Picture Concepts	36	N	Block design test	36	N
	Story recall	36	N	Digit Symbol coding	36	N						
	Phonological Verbal Fluency	36	Y	Picture Concepts	36	N						
	Picture Concepts	36	N									
	Corsi Block tapping	36	N									
Sreenivasan et al.^49^	Digit span	22	Y									
Zanaboni et al.^50^	BRIEF	129	–	BRIEF	129	–	BRIEF	129	–	BRIEF	129	–
Sousa et al.^51^	Epitrack Junior	18	N	Epitrack Junior	18	N	Epitrack Junior	18	N	Epitrack Junior	18	N
Wu et al.^52^				Attention network test (ANT)	42	Y						
Shi et al.^53^				Eye tracking assessment	36	Y	Eye tracking assessment	36	Y			
Duma et al.^18^	Digit Span Test	13	N^a^	Conners continuous performance test	13	N^a^	Phonological verbal fluency	13	N^a^	Complex Figure of Rey	13	N^a^
	Phonological Verbal Fluency	13	N^a^	Phonological verbal fluency	13	N^a^	Tower of London	13	N^a^	Tower of London	13	N^a^
	Corsi block tapping test	13	N^a^	Tower of London	13	N^a^						
Ragab et al.^54^	Digit Span Test	22	Y				Trail‐making test	22	Y	Complex Figure of Rey	22	Y
							Wisconsin Card Sorting Test	22	Y			
							COWAT	22	Y	COWAT	22	Y
Chen et al.^55^	Digit Span Test	26	N^b^				Wisconsin Card Sorting Test	26	N^b^			

Abbreviations: BRIEF, Behavior rating inventory of executive function; CANTAB, Cambridge Neuropsychological Test Automated Battery; COWAT, Controlled Oral Word Association Test; D‐KEFS, Delis–Kaplan Executive Function System Battery; KET‐KID, Cognitive Developmental Scale for Preschool Children; MFFT, Matching Familiar Figures Test; N, no; NEPSY‐II, NEuroPSYchological Assessment – Second Edition; RAVLT, Rey Auditory Verbal Learning Test; TOMAL, Test of Memory and Learning; Y, yes; WRAML, Wide Range Assessment of Memory and Learning.

^a^ Compared with self‐limited epilepsy with autonomic seizures patients.

^b^ Compared with childhood absence epilepsy patients.

In total, 43 different tests were used to assess EFs across all studies. The “digits span” was used in 16 studies^16^, ^17^, ^18^, ^26^, ^28^, ^29^, ^32^, ^33^, ^38^, ^42^, ^43^, ^47^, ^48^, ^49^, ^54^, ^55^ (39.0%); the “Phonological Verbal Fluency” in 12 studies^16^, ^17^, ^21^, ^24^, ^31^, ^32^, ^37^, ^44^, ^45^, ^47^, ^48^, ^55^ (29.3%); the “Trail‐Making test” in 10 studies^16^, ^17^, ^20^, ^21^, ^24^, ^28^, ^29^, ^31^, ^32^, ^54^ (24.4%); and the “Wisconsin card sorting test” in eight studies^21^, ^27^, ^28^, ^32^, ^39^, ^54^, ^55^, ^56^ (19.5%). The “Complex Figure of Rey” was used in six studies^16^, ^17^, ^18^, ^31^, ^48^, ^54^ (14.6%). The “Rey Auditory Verbal Learning test,”^16^, ^17^, ^31^, ^48^ the “Corsi block tapping test,”^18^, ^44^, ^47^, ^48^ the “Digit Symbol — Coding,”^22^, ^29^, ^35^, ^48^ and the “Tower of London”^16^, ^17^, ^18^, ^40^ were each used in four studies (9.8%).

The “Stroop test,”^33^, ^39^, ^40^ the “Behavior rating inventory of executive function‐(BRIEF) questionnaire,”^30^, ^34^, ^50^ the “Delis–Kaplan Executive Function System Battery,”^23^, ^34^, ^35^ the “Wide Range Assessment of Memory and Learning,”^21^, ^28^, ^32^ the “Story recall test,”^16^, ^17^, ^48^ the “Semantic verbal fluency,”^44^, ^45^, ^48^ and the “block span”^16^, ^17^, ^47^ were each used in three studies (7.3%).

The “Attention network test,”^25^, ^52^ the “Picture Concepts,”^32^, ^48^ the “block design test,”^29^, ^48^ the “Letter‐Number sequencing,”^32^, ^43^ the “Spatial Learning test,”^16^, ^17^ the “controlled oral word association test,”^40^, ^54^ the “Children's Memory Scale,”^22^, ^35^ and the “Conners continuous performance test”^18^, ^38^ were used in two studies each (4.9%).

Finally, the “Eye tracking assessment,”^53^ the “Go/No‐Go Task,”^41^ the “Epitrack Junior,”^51^ the “Test of Memory and Language,”^37^ the “Verbal memory screening test,”^45^ the “CANTAB Spatial Working Memory,”^36^ the “NEPSY‐II,”^46^ the “five point test,”^26^ the “Similarities,”^32^ the “Letter cancellation test,”^29^ the “Spatial Memory Test,”^29^ the “Matching Familiar Figures Test,”^28^ the “Sternberg maintenance task,”^42^ the “Everyday Attention for Children test,”^23^ the “Cognitive Developmental Scale for Preschool Children,”^19^ the “Modified card sorting test,”^40^ the “Alpha span test,”^26^ and the “Test of Memory and Learning”^37^ were each employed in one study (2.4%).

### 3.4 Working memory

A total of 22 distinct neuropsychological tests were used to assess working memory (Table 2). Altogether, these tests were carried out 1453 times in patients with SeLECTS in comparison with healthy controls and showed abnormal findings in 667 instances (45.9%). Working memory was impaired in 253 (40.7%) of the 621 patients with SeLECTS in whom it was evaluated when compared with healthy controls, regardless of which test was employed.

### 3.5 Inhibition

Authors investigated inhibitory control using 21 different neuropsychological tools (Table 2), which were administered a total of 1175 times in patients with SeLECTS compared with healthy controls, yielding abnormal results in 807 (68.7%). Inhibition ability was compromised in 590 (73.1%) of the 807 patients in whom it was evaluated, irrespective of the specific test used.

### 3.6 Cognitive flexibility

Cognitive flexibility was assessed in patients with SeLECTS through 14 different tests (Table 2) in 1155 occasions, with abnormal results recorded in 774 cases (67.0%). This function was affected in 406 out of 642 patients with SeLECTS (63.2%) in whom it was assessed compared with healthy controls.

### 3.7 Higher order EFs

Of the 41 studies reviewed, 10 incorporated higher order EFs (planning and problem‐solving) assessments, using seven distinct tests (Table 2) administered 302 times overall, with 181 pathological results (59.9%). Of the 232 individuals tested for these specific functions, 133 (57.3%) exhibited poorer performances in comparison to healthy controls, regardless of the assessment tool used.

### 3.8 EFs evaluation in patients with SeLECTS compared with other clinical populations

In 10 studies, in addition to healthy controls, authors included comparison groups composed of patients diagnosed with different types of epilepsy, including idiopathic generalized epilepsies and other forms of self‐limited focal epilepsy.

In a study^55^ involving 26 patients with SeLECTS, authors assessed cognitive abilities with the Raven's Progressive Matrices, working memory with the Digit Span Test, and cognitive flexibility with the Wisconsin Card Sorting Test (WCST), and compared the results with those of a control group formed by 10 patients diagnosed with childhood absence epilepsy (CAE), finding no significant differences. Likewise, in a study^18^ including 13 patients with SeLECTS, cognitive abilities (Wechsler Intelligence Scales), working memory (Digit Span Test, Phonological Verbal Fluency Test, and Corsi Block Tapping Test), inhibition (Phonological Verbal Fluency Test, Tower of London, and Conners' Continuous Performance Test), cognitive flexibility and planning (Phonological Verbal Fluency Test, Tower of London) were evaluated in comparison with a control group of eight patients diagnosed with self‐limited epilepsy with autonomic seizures (SeLEAS), with no significant discrepancies between groups.

### 3.9 Correlations between EFs measurements and clinical data

Across the selected studies, executive dysfunction was linked to multiple clinical variables, yielding varied and sometimes contrasting findings.

#### 3.9.1 Age at seizure onset

Neri et al.^21^ showed that children with later onset performed better on the WRAML (Wide Range Assessment of Memory and Learning) Digital Windows subtest (Mann–Whitney test, *p* = 0.010); Malfait et al.^23^ found correlations with phonological verbal fluency (*r* = 0.555, *p* = 0.032) and with Stroop errors (*r* = 0.803, *p* < 0.001); Yang et al.^25^ reported associations with ANT (attention network test) accuracy (β = 1.49, SE = 0.33, *p* < 0.001) and ANT grand mean effect (β = −34.23, SE = 8.48, *p* < 0.001); Elkholy et al.^29^ described correlations with trail making, letter cancellation and RT scores, and a significant correlation with correct responses (*p* = 0.036); Ayaz et al.^39^ found a correlation with Stroop total score (*r* = −0.434, *p* = 0.015),^55^ and Wu et al.^52^ with ANT accuracy (*r* = 0.730, *p* < 0.001; *r* = 0.330, *p* = 0.033 at follow‐up) and ANT executive control network (*r* = 0.369, *p* = 0.019). Shi et al.^53^ demonstrated a strong correlation with the antisaccade task (*r* = −0.613, *p* < 0.0001).

#### 3.9.2 Seizure frequency

Ayaz et al.^39^ described a direct correlation with Stroop total score (*r* = 0.501, *p* = 0.004); Wu et al.^52^ reported associations with ANT accuracy both at baseline and after a 7‐year follow‐up (*r* = 0.503, *p* = 0.001; *r* = 0.366, *p* = 0.017 at follow‐up) and the ANT executive control network (*r* = 0.373, *p* = 0.015); Shi et al.^53^ confirmed a correlation with antisaccade task (*r* = 0.399, *p* = 0.016).

#### 3.9.3 Interictal epileptiform abnormalities

Yang et al.^25^ found that NREM spike index correlated inversely with ANT accuracy (β = −0.07, SE = 0.03, *p* = 0.016), while Vintan et al.^36^ observed a correlation between centro‐temporal spikes frequency and SSP span length (*U* = 18.5, *p* < 0.05).

#### 3.9.4 Pharmacological treatment

Wu et al.^52^ described a correlation between longer treatment duration and lower ANT accuracy (*r* = 0.500, *p* < 0.001), while Ayaz et al.^39^ found a direct correlation with Stroop total score (*r* = 0.364, *p* = 0.044).

#### 3.9.5 Epilepsy duration

Malfait et al.^23^ reported that longer active disease correlated with poorer phonological verbal fluency (*r* = −0.620, *p* = 0.014), and Teixeira et al.^45^ confirmed this finding through regression analysis.

#### 3.9.6 Seizure type

Neri et al.^21^ observed that children with focal seizures outperformed those with generalized seizures in the WCST failure to maintain set (*p* = 0.037).

### 3.10 Psychopathological assessment

The instruments used to assess psychopathological dimensions in the included studies, together with the corresponding results, are detailed in Table S1. The Child Behavior Checklist (CBCL) was employed in five studies^24^, ^30^, ^39^, ^51^, ^54^; The Kiddie Schedule for Affective Disorders and Schizophrenia (K‐SADS) was applied in three studies^39^, ^54^, ^57^; the Conners Rating Scales (CRS) were applied in three studies^43^, ^58^, ^59^; the Attention Deficit Hyperactivity Disorder Symptom Checklist‐4 (ADHD‐SC4),^60^ the Structured Clinical Interview for the DSM‐IV‐TR,^61^ the Strengths and Difficulties Questionnaire,^59^ the Barratt Impulsiveness Scale–11,^59^ and the Faux‐Pas Child Task (FP)^61^ were used in one study each.

Kagitani‐Shimono et al.^30^ reported significantly higher internalizing (*p =* 0.004), externalizing (*p =* 0.008), and total (*p =* 0.005) scores in patients with SeLECTS compared with healthy controls. Likewise, Ayaz et al.^39^ found significantly higher externalizing (*p =* 0.025) and overall (*p =* 0.033) scores in the SeLECTS group compared with controls. Ragab and colleagues^54^ investigated patients with SeLECTS alongside those with other epileptic syndromes—idiopathic childhood occipital epilepsy of Gastaut (ICOE‐G) and SeLEAS, formerly known as Panayiotopoulos syndrome—and observed that all three groups, when compared with healthy controls, showed abnormalities across multiple domains, including anxiety–depression (*p =* 0.001), withdrawn–depression (*p =* 0.007), somatic complaints (*p =* 0.001), social problems (*p =* 0.001), thought problems (*p =* 0.001), attention problems (*p =* 0.001), internalizing difficulties (*p =* 0.001), externalizing difficulties (*p =* 0.041), total problems (*p =* 0.001), total competence (*p =* 0.001), and sluggish cognitive tempo (*p =* 0.001). Xiao et al.^24^ examined potential associations between psychopathological dimensions and brain network parameters (nodal metrics) in a SeLECTS cohort, and found that the nodal degree of the right postcentral gyrus was negatively correlated with attention problems (*p =* 0.004) and aggressive behavior (*p =* 0.002), while the nodal degree of the bilateral postcentral gyri showed negative correlations with delinquent behavior (left: *p =* 0.002; right: *p =* 0.001). Moreover, a reduced nodal degree in the left postcentral gyrus was linked to higher attention problems (*p =* 0.017) and increased aggressive behavior (*p =* 0.010). In contrast, Sousa et al.^51^ reported no significant differences in psychopathological and emotional dimensions between patients with SeLECTS and healthy controls.

Ayaz et al.^39^ identified a significantly higher prevalence of psychological disorders in patients with SeLECTS compared with healthy controls (*p =* 0.021); Ragab and colleagues^54^ reported rates of 59.1% for attention deficit hyperactivity disorder (ADHD), 50.0% for depression, 50.0% for anxiety, 22.7% for oppositional–defiant disorder (ODD), and 13.6% for conduct disorder among patients with SeLECTS, with each condition occurring at a significantly higher frequency than in controls (*p =* 0.001); Orak et al.^57^ identified an ADHD diagnosis in 28.0% of the SeLECTS sample.

Ciumas et al.^43^ observed significantly elevated hyperactivity/impulsivity (*p =* 0.004) and ADHD index scores (*p =* 0.001) in patients with SeLECTS compared with healthy controls; in contrast, Smith et al.^58^ reported no significant differences between patients with SeLECTS and healthy controls, either at baseline or at the final assessment conducted after a mean follow‐up of 4.9 years; similarly, Tin et al.^59^ found no statistically significant differences between patients with SeLECTS and controls.

Lima et al.^61^ reported that 60.7% of patients with SeLECTS had a psychiatric disorder, including 52.1% with ADHD and 26.1% with anxiety disorders; however, no comparison with healthy controls was carried out.

In one retrospective study,^60^ the prevalence of ADHD in patients with SeLECTS was 72.5%, with the combined subtype identified in 45.0%.

Social cognition was assessed by Lima et al.^61^ who reported a significant overall impairment in patients with SeLECTS compared with healthy controls (*p <* 0.01).

Impulsivity and related behavioral features were examined in a single study,^59^ describing significantly higher impulsivity (*p =* 0.038) only in patients with SeLECTS who experienced seizures after bedtime, whereas those with seizures clustered in the pre‐waking phase did not differ from healthy controls.

### 3.11 Correlations between EFs measurements and psychopathological and emotional dimensions

Only one study by Lima et al.^61^ explored potential correlations between social cognition performance, measured by the FP test, and standard EFs. The authors used Pearson and Kendall correlation within the SeLECTS cohort and identified associations between FP alterations and impairments in tests assessing working memory, mental abstraction, inhibition, and cognitive flexibility (Table S1).

### 3.12 Patient and caregiver‐oriented questionnaires

The measures employed to evaluate daily functioning across the included studies, along with their respective findings, are reported in Table S1. The School Performance Test (SPT),^20^ the Ansula Behavior Rating Scale^17^ (a shortened version of the Anser System^62^), the Pediatric Quality of Life Inventory (PedsQL)^50^ along with parent‐ and teacher‐based questionnaires^16^ (adopted from a previous publication^63^), were used in one study each.

Miziara et al.^20^ found that patients with SeLECTS reported poorer school performance than healthy controls (*p =* 0.008), with parents' (*p =* 0.004) and teachers' (*p =* 0.008) reports consistent with this observation. Lindgren et al.^17^ found no significant differences between patients with SeLECTS and healthy controls based on reports from parents, teachers, or the children themselves regarding everyday behavioral functioning. Croona et al.^16^ observed greater difficulties among patients with SeLECTS compared with healthy controls in distractibility (*p <* 0.05), concentration (*p <* 0.01), temper (*p <* 0.001), impulsiveness (*p <* 0.05), and ability to follow instructions (*p <* 0.05) according to parents, as well as poorer reading comprehension (*p <* 0.05) according to teachers. Focusing on their cohort of patients with SeLECTS, Zanaboni et al.,^50^ reported that the children's health‐related quality of life (HRQoL) was generally in the average range.

### 3.13 Correlations between EFs measurements and daily functioning

Only one study, by Zanaboni et al.,^50^ investigated the relationship between executive functions and daily functioning. Using the Pediatric Quality of Life Inventory (PedsQL), a patient‐ and caregiver‐reported measure of quality of life, and the BRIEF questionnaire to assess executive functioning, the authors observed that lower executive functioning was correlated with lower overall quality of life scores (Table S1).

### 3.14 Proposal for a standardized EFs evaluation protocol

**FIGURE 2:**
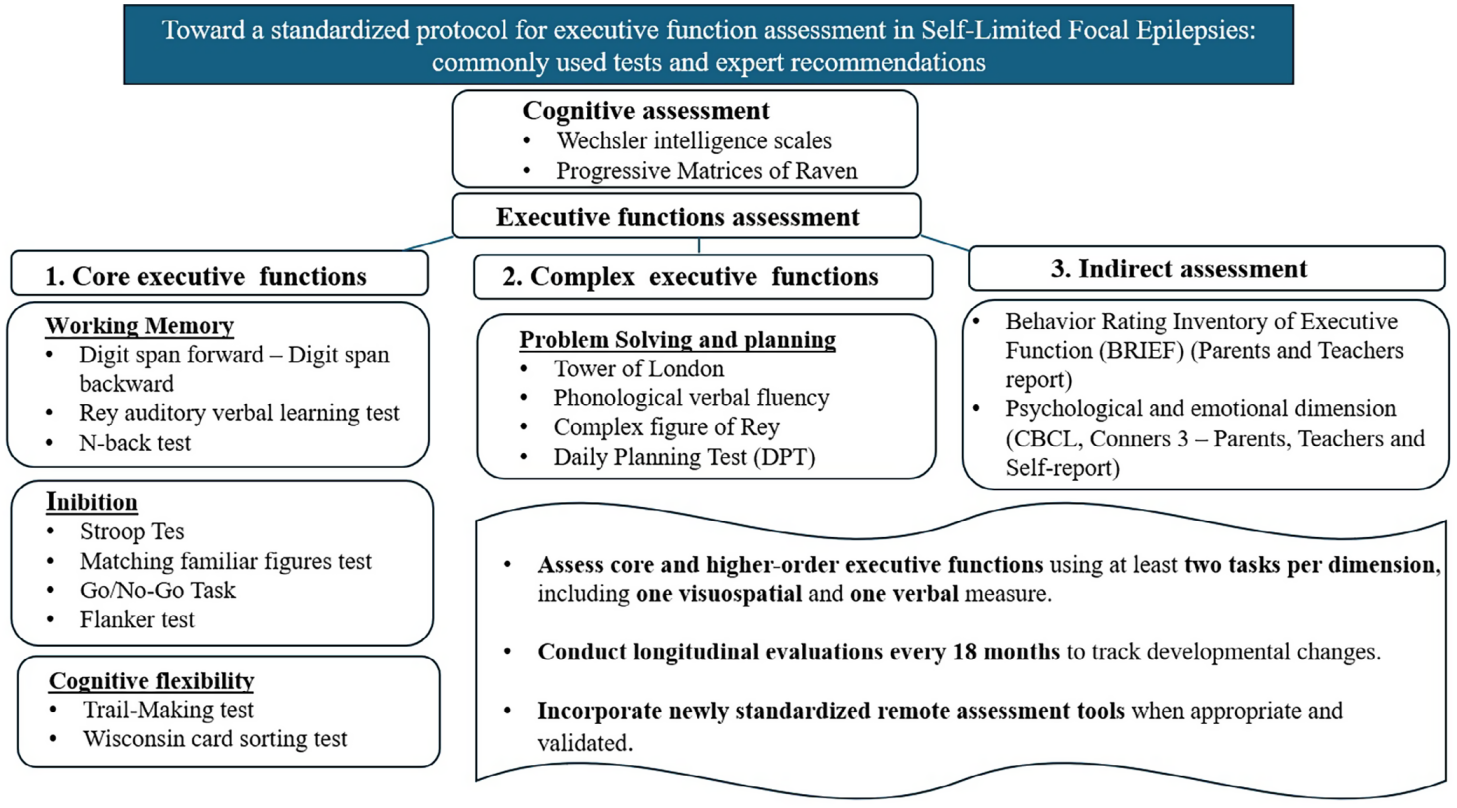
Proposal for a protocol to assess executive functions in self‐limited focal epilepsies.

Based on our results and taking into account the high methodological variability across the examined studies, we may propose a standardized protocol to assess executive functioning, psychopathological and emotional dimensions, and daily functioning in patients with SeLECTS, integrating the most used assessment tools with expert‐recommended measures, such as the Daily Planning Test (DPT) for complex EFs. As illustrated in Figure 2, our protocol includes both verbal and nonverbal measures, indirect examination of executive functioning that might allow for a more sensitive detection of EFs difficulties. Furthermore, we suggest the use of patient and caregivers‐oriented questionnaires as well as similar instruments for psychopathological and emotional dimensions evaluation. In this framework, longitudinal monitoring is essential, including periodic reassessments at 18‐month intervals.

## 4 DISCUSSION

The present scoping review analyzed the current knowledge on EFs in patients with SeLECTS. Our study updates two recent systematic reviews on the topic.^6^, ^7^ Ramos et al.^6^ concluded their systematic review and meta‐analysis of 19 articles by reporting deficits in EFs—specifically in the domains of inhibitory control, cognitive flexibility, and verbal fluency—in 466 out of 561 (83.1%) SeLECTS patients compared with control groups. However, the quality of the evidence was rated as very low, leading the authors to suggest caution in the interpretation of these findings. Zanaboni et al.,^7^ in their systematic review of 23 articles, described executive dysfunction in 417 out of 636 (65.6%) patients with SeLECTS, particularly in the domains of inhibition and cognitive flexibility.

In this scoping review, we found that despite the favorable prognosis in terms of seizure remission, a significant percentage of children with SeLECTS might exhibit a complex pattern of neuropsychological deficits, with a particular involvement of EFs.

General cognitive abilities were within the normal range for all patients with SeLECTS studied. However, in 22.9% of patients overall IQ scores assessed using Wechsler scales were significantly lower compared with healthy controls, as reported in seven studies.^30^, ^31^, ^39^, ^40^, ^43^, ^46^, ^53^

Despite the substantial heterogeneity in the neuropsychological tools adopted, the total of test measurements assessing working memory revealed altered results in 45.9% of the instances. Among the tests exploring inhibition, altered values were observed in 68.7% of combined administrations, while putting together tools evaluating cognitive flexibility, the percentage of pathological findings was 67.0%, and for those assessing planning and problem‐solving abilities, the rate amounted to 59.9%. While our findings concerning inhibitory control and cognitive flexibility align with those reported by Ramos et al.^6^ and Zanaboni et al.,^7^ we strengthen the occurrence of a consistent impairment in working memory and in higher order EFs.

Ramos et al.^6^ analyzed pooled data by eight studies, selected as eligible for meta‐analysis of working memory deficits and did not find statistically significant differences between the performance of children with SeLECTS and healthy controls. However, the authors emphasized considerable heterogeneity across the studies, cautioning that these findings should be interpreted with care; they also noted a possible underestimation bias, as some tests might lack sufficient sensitivity to detect working memory deficits. On the other hand, in the systematic review by Zanaboni et al.,^7^ a clear difference between patients and controls emerged in only one study,^17^ hence the role of working memory was not further investigated.

Our scoping review, based on a comprehensive analysis of the information obtained from more recent studies, combined with those extracted by the aforementioned systematic reviews, strongly suggests a negative effect of SeLECTS on working memory.

Regarding higher order EFs, Ramos et al.^6^ did not perform a meta‐analysis for this topic due to insufficient number of eligible studies. Even so, the authors inferred a possible underestimation of higher order EFs deficits, since they identified impairments in inhibition and cognitive flexibility—core components of higher level executive processes—in subjects with preserved higher order EFs. Zanaboni et al.^7^ identified five studies that assessed these functions, revealing significantly poorer performance in patients with SeLECTS compared with healthy controls in two. Here, we delineated impairments in over half (57.3%) of patients with SeLECTS with high‐order EFs impairment, which is corroborated by the occurrence of deficits in basic EFs, the essential foundation of higher level processes.

Additionally, we found that in most cases, each EF subcategory was evaluated through the administration of multiple neuropsychological tools to the same group of patients (e.g., digit span, block span, Rey Auditory Verbal Learning Test, story recall, spatial learning test, and phonological verbal fluency to assess working memory within a single study). This practice might introduce confirmation bias, as discrepant results can be observed for the same function within the same sample.

As emerged by this study, EFs in patients with SeLECTS have also been evaluated in relation to other types of epilepsy. Chen et al.^55^ compared working memory and cognitive flexibility between patients with SeLECTS and those with CAE, while Duma and coworkers^18^ examined working memory, inhibitory control, cognitive flexibility, and planning abilities in patients with SeLECTS in comparison with individuals diagnosed with SeLEAS. In both cases, performances did not differ significantly between groups, suggesting that such EFs impairments might similarly occur in other forms of self‐limited focal epilepsy or idiopathic generalized epilepsy. Of note, no comparison has been carried out with structural epilepsies involving fronto‐temporal areas that are well known to be implicated in EFs.

Zanaboni and co‐workers^7^ also examined the relationship between EFs impairment and clinical characteristics of patients with SeLECTS such as epileptic activity, interictal EEG activity, and seizure frequency. These authors correlated executive dysfunctions with an earlier age at epilepsy onset and with a higher frequency of interictal epileptic activity during NREM sleep. Conversely, they found no correlation between EFs impairments and either ASM treatment or the lateralization of EEG interictal epileptic activity.

By incorporating both earlier and more recent studies, our scoping review confirmed the previously reported association between EFs and earlier age at onset, and the likely detrimental effect on EFs development when epileptic activity begins earlier. We also confirmed the negative effect of interictal epileptic activity during NREM sleep on executive functioning. In addition, we retrieved three studies correlating EFs alteration with higher seizure frequency.^18^, ^43^, ^55^

Consistently with Zanaboni et al.,^7^ we found no correlations between EFs and the lateralization of seizure onset zone on the EEG across the included studies.

With regards to pharmacological treatment, we identified two studies reporting EFs impairment in patients treated with ASM, one included by Ramos and co‐workers^6^ that is, Ayaz et al.,^39^ and one released subsequently by Wu et al.^52^ Conversely, Zanaboni and co‐authors^7^ included one study in which no differences were observed between treated and nontreated patients with SeLECTS. Due to the heterogeneity in the treatment of patients with SeLECTS and the low number of studies addressing this topic, it is not possible to attribute a causal role to the treatment itself. As a confounding factor, clinicians typically start ASM in patients with SeLECTS that manifest those clinical features that were described in association with greater executive dysfunction (i.e., earlier epilepsy onset, higher frequency of epileptiform abnormalities and higher seizure burden).

As additional clinical variable, Neri et al.^21^ reported that patients with focal seizures outperformed those with generalized seizures on measures of cognitive flexibility. The association between executive deficits and an increased tendency for epileptic activity to spread to brain regions other than fronto‐temporal cortex could further strengthen the correlation between epileptic activity and executive dysfunction.

Also, we analyzed a recent study^54^ that conducted a comprehensive sleep assessment using polysomnography and reported no correlation with executive dysfunction.

Assessments of psychopathological or emotional dimensions and their potential correlations with executive functioning were reported in 11 studies.^24^, ^30^, ^39^, ^43^, ^51^, ^54^, ^57^, ^58^, ^59^, ^60^, ^61^ Five of these studies^24^, ^51^, ^57^, ^58^, ^60^ described the presence of clinical psychopathological diagnoses, but did not perform any comparison with control groups, whereas the remaining six^30^, ^39^, ^43^, ^54^, ^59^, ^61^ identified a significantly higher prevalence of these disorders in patients with SeLECTS compared with healthy controls. Among the reported diagnoses, ADHD was the most frequent, with a prevalence ranging between 16.6% and 75.5%. In addition, one study^61^ described a correlation between EF deficits and impairments in theory of mind. This complex mental ability, essential for effective social interaction, is required to predict, interpret, and explain relevant actions and behaviors, and relies on multiple EFs resources.

In addition, we could examine daily functioning through patient‐ and caregiver‐oriented questionnaires in four studies.^16^, ^17^, ^20^, ^50^ Specifically, three studies^16^, ^17^, ^20^ used questionnaires on school performance, with two^16^, ^20^ reporting greater difficulties in patients with SeLECTS compared with controls. The remaining study^50^ enrolling only patients with SeLECTS reported that lower functioning in EFs was associated with poorer quality of life.

Finally, based on this review and expert opinions on the topic, we developed a proposal for a standardized EFs and multidimensional assessment protocol for patients with SeLECTS. The protocol includes a targeted assessment of all core EFs (i.e., inhibition, working memory, cognitive flexibility) as well as complex EFs (i.e., problem‐solving and planning) through at least two tasks per EFs' domain, encompassing both verbal and visuospatial measures. Although this approach might appear highly demanding and potentially redundant, it may be adequate for patients with SeLECTS, in whom EF alterations are often subtle and both verbal and visuospatial abilities might be only mildly impaired.^64^

In this protocol, we also included an indirect assessment of executive functioning through parent‐report questionnaires, which may capture executive behaviors in everyday life contexts and be more sensitive in identifying vulnerabilities in complex real‐world demands (e.g., school‐related activities) than standard laboratory‐based assessments.^65^, ^66^

In addition, the protocol incorporates an evaluation of psychopathological and emotional dimensions.

Given the developmental trajectory of SeLECTS and the progressive maturation of executive functions, our multidimensional assessment should also be implemented within a longitudinal framework. This approach would allow a more comprehensive understanding of the evolution of executive vulnerabilities and their relationship with changing environmental demands across development,^66^ the course of epilepsy and changes in pharmacological treatment.

In this regard, the use of newly standardized remote assessment tools, when appropriate and validated,^67^ should be encouraged in this clinical population which usually does not require hospitalization.

## 5 CONCLUSIONS

Our scoping review confirms and expands the knowledge that children with SeLECTS might exhibit EFs impairments, involving both basic and higher order EFs.

These disorders appear to be modulated by clinical variables such as age at epilepsy onset, epilepsy duration, and seizure frequency. The role of ASM appears more controversial due to the heterogeneity in type of treatment across studies and additional clinical factors possibly influencing the timing of initiating it.

However, significant alterations in global cognitive and academic performance are rarely seen in patients with SeLECTS. This dissonance could be explained by different factors, including the self‐limiting nature of this epilepsy, individual variability in the severity of deficits, alternative cognitive strategies, and the use of standard cognitive and scholastic assessments lacking sensitivity to detect subtle EF abnormalities.

From a clinical perspective, our findings highlight the need for tailored and longitudinal neuropsychological monitoring in children with SeLECTS, particularly those experiencing frequent seizures or overt behavioral abnormalities. Early identification of executive dysfunction might prompt timely psychoeducational interventions in children at higher risk for possible negative consequences regarding school performance and social achievements.

Moreover, the considerable variability observed in the methods used to assess executive functions underscores the need for a more standardized approach.

For these reasons, by integrating the evidence from our review with expert opinions, we developed a proposal for a standardized protocol for the assessment of EFs in patients with SeLECTS, incorporating psychopathological and emotional dimensions as well as daily functioning.

Further longitudinal studies are needed to better clarify the possible impact of EFs impairment in children with SeLECTS and in general with self‐limiting epilepsies.

## FUNDING INFORMATION

This publication was produced with the co‐funding of the European Union—Next Generation EU, in the context of The National Recovery and Resilience Plan, Investment 1.5 Ecosystems of Innovation, Project Tuscany Health Ecosystem (THE), ECS00000017. Spoke 3. CUP: B83C22003920001.

**Test Yourself**

1. Based on this scoping review, which executive functions are reported to be mostly affected in children with SeLECTS?
  - Only emotional regulation
  - Only basic executive functions
  - Both basic and high‐order executive functions
  - Only attention and memory
  - Only language‐related functions
2. What clinical recommendation is suggested based in the results of this review?
  - Avoid the use of ASM in children with SeLECTS
  - Delay treatment until cognitive decline is evident
  - Provide standardized education plans for all children with epilepsy
  - Implement tailored neuropsychological monitoring for at‐risk children
  - Refer all children with SeLECTS for surgical intervention
3. Why might standard cognitive and academic assessments fail to detect EFs impairment in children with SeLECTS?
  - They are not administered frequently enough
  - They may lack sensitivity to subtle EFs deficits
  - They are too difficult for children with epilepsy
  - They focus on behavioral problems only
  - They are influenced by socioeconomic background rather than medical history

*Answers may be found in the* *Supporting information*.

## Supporting information


Table S1.

Data S1.

## Data Availability

Data sharing not applicable to this article as no datasets were generated or analyzed during the current study.
