# Supplementary material for: Executive functions and self‐limited epilepsy with centro‐temporal spikes: A scoping review
Source: Epileptic Disord. 2026 Jan 12;28(2):275–94. doi: 10.1002/epd2.70176 (PMC13084210; doi:10.1002/epd2.70176)
Supplement: Supplementary file 1 — Table S1. [file EPD2-28-275-s001.docx]

| **Supplementary Table 1-Psychopathological and emotional dimensions** | | | | | | |
| --- | --- | --- | --- | --- | --- | --- |
| Study | Assessment tool | Findings in SeLECTS patients | Comparison with HC | Statistics | Correlations with EFs | EFs assessment (statistics) |
| Ayaz et al.^44^ | CBCL | Internalizing problems | No significant difference | *p = 0.070* | NA | - |
|  |  | Externalizing problems | Statistically higher in SeLECTS | *p = 0.025* | NA | - |
|  |  | Total problems | Statistically higher in SeLECTS | *p = 0.033* | NA | - |
|  | K-SADS | At least one diagnosis in 65.5%  ADHD = 25.8%  Specific phobia = 16.1%  Sleep disorder = 12.9%  Enuresis = 9.7%  ODD = 9.7%  SLD = 9.7%  Tic disorder = 6.7%  MDD = 6.5%  Speaking disorder = 3.2%  Encopresis = 3.2%  SAD = 3.2%  Social phobia = 3.2% | Statistically higher in SeLECTS (numbers of subjects with at least one diagnosis) | *p = 0.021* | NA | - |
| Ragab et al.^25^ | CBCL | Anxiety–depression | Statistically higher in SeLECTS | *p = 0.001* | NA | - |
|  |  | Withdrawn–depression | Statistically higher in SeLECTS | *p = 0.007* | NA | - |
|  |  | Somatic complaints | Statistically higher in SeLECTS | *p = 0.001* | NA | - |
|  |  | Social problems | Statistically higher in SeLECTS | *p = 0.001* | NA | - |
|  |  | Thought problems | Statistically higher in SeLECTS | *p = 0.001* | NA | - |
|  |  | Attention problems | Statistically higher in SeLECTS | *p = 0.001* | NA | - |
|  |  | Internalizing problems | Statistically higher in SeLECTS | *p = 0.001* | NA | - |
|  |  | Externalizing problems | Statistically higher in SeLECTS | *p = 0.041* | NA | - |
|  |  | Total problems | Statistically higher in SeLECTS | *p = 0.001* | NA | - |
|  |  | Total competence | Statistically higher in SeLECTS | *p = 0.001* | NA | - |
|  |  | Sluggish cognitive tempo | Statistically higher in SeLECTS | *p = 0.001* | NA | - |
|  | K-SADS | ADHD = 59.1%  MDD = 50.0%  Anxiety = 50.0%  ODD = 22.7%  Conduct disorder = 13.6% | All Statistically higher in SeLECTS | *p = 0.001* | NA | - |
| Kagitani-Shimono et al.^16^ | CBCL | Internalizing problems | Statistically higher in SeLECTS | *p = 0.004* | NA | - |
|  |  | Externalizing problems | Statistically higher in SeLECTS | *p = 0.008* | NA | - |
|  |  | Total problems | Statistically higher in SeLECTS | *p = 0.005* | NA | - |
| Xiao et al.^43^ | CBCL | Comparison with brain network parameters (nodal metrics) only | NA | *-* | NA | - |
| Sousa et al.^55^ | CBCL | Internalizing problems | No significant difference | *p = 0.165* | NA | - |
|  |  | Externalizing problems | No significant difference | *p = 0.201* | NA | - |
|  |  | Total problems | No significant difference | *p = 0.450* | NA | - |
| Orak et al.^57^ | K-SADS | ADHD = 28.0% | NA | *-* | NA | - |
| Ciumas et al.^29^ | CRS | Hyperactivity/impulsivity | Statistically higher in SeLECTS | *p=0.003* | NA | - |
|  |  | Psychosomatic | No significant difference | *p = 0.100* | NA | - |
|  |  | Learning Problems | No significant difference | *p=0.005* | NA | - |
|  |  | Indices of ADHD | Statistically higher in SeLECTS | *p = 0.001* | NA | - |
|  |  | Anxiety | No significant difference | *p = 0.300* | NA | - |
|  |  | Conduct Disorder | No significant difference | *p = 0.009* | NA | - |
| Smith et al.^58^ | CRS | DCD = 50.0% (basal), 25.0% (4.5 years follow-up) | No significant difference | NA | NA | - |
|  |  | DD = 25.0% (basal), 33.0% (4.5 years follow-up) | No significant difference | NA | NA | - |
|  |  | ADHD = 16.6.% (basal), 8.3% (4.5 years follow-up) | No significant difference | *p = 0.290* | NA | - |
| Tin et al.^59^ | CRS | ADHD Index | No significant difference  (seizures close to bedtime) | *p = 0.435* | NA | - |
|  |  |  | No significant difference  (seizures close to awakening) | *p = 0.528* |  |  |
|  |  | Oppositional subscale | No significant difference  (seizures close to bedtime) | *p = 0.350,* |  |  |
|  |  |  | No significant difference  (seizures close to awakening) | *p = 0.741* | NA | - |
|  |  | Hyperactivity subscale | No significant difference  (seizures close to bedtime) | *p = 0.479* | NA | - |
|  |  |  | No significant difference  (seizures close to awakening) | *p = 0.799* |  |  |
|  |  | Cognitive problem  subscale | No significant difference  (seizures close to bedtime) | *p = 0.815* |  |  |
|  |  |  | No significant difference  (seizures close to awakening) | *p = 0.651* | NA | - |
|  | Strengths and Difficulties Questionnaire | Overall stress | No significant difference  (seizures close to bedtime) | *p = 0.325* | NA | - |
|  |  |  | No significant difference  (seizures close to awakening) | *p = 0.727* |  |  |
|  |  | Behavioral difficulties | No significant difference  (seizures close to bedtime) | *p = 0.016,* |  |  |
|  |  |  | No significant difference  (seizures close to awakening) | *p = 0.578* | NA | - |
|  |  | Hyperactivity and  concentration difficulties | No significant difference  (seizures close to bedtime) | *p = 0.324* | NA | - |
|  |  |  | No significant difference  (seizures close to awakening) | *p = 0.466* |  |  |
|  |  | Emotional distress | No significant difference  (seizures close to bedtime) | *p =975* |  |  |
|  |  |  | No significant difference  (seizures close to awakening) | *p = 0.974* | NA | - |
|  |  | Difficulties getting along  with other children | No significant difference  (seizures close to bedtime) | *p=0.363,* | NA | - |
|  |  |  | No significant difference  (seizures close to awakening) | *p =0.404* |  |  |
|  |  | Kind and helpful behavior | No significant difference  (seizures close to bedtime) | *p=0.012* |  |  |
|  |  |  | No significant difference  (seizures close to awakening) | *p = 0.295* | NA | - |
|  | Barratt Impulsiveness Scale–11 | Impulsiveness | Statistically higher in SeLECTS (seizures close to bedtime) | *p=0.038* | NA | - |
|  |  |  | No significant difference  (seizures close to awakening) | *p = 0.203* |  |  |
| Lima et al.^61^ | Structured Clinical Interview for the DSM-IV-TR | At least one diagnosis in 60.7%  ADHD = 52.1%  Anxiety = 26.1% | NA | *-* | NA | - |
|  | Faux-Pas Child Task | Social cogniton | Statistically higher in SeLECTS | *p < 0.01* | Working memory | Digits Total  r = 0.411  p = 0.050 |
|  |  |  |  |  |  | Finger Windows  r = 0.518  p = 0.014 |
|  |  |  |  |  |  | Number and Letter Sequence  r = 0.630  p = 0.001 |
|  |  |  |  |  | Mental abstraction | Picture Concepts  r = 0.534  p = 0.009 |
|  |  |  |  |  |  | Similarities  r = 0.712  p < 0.001) |
|  |  |  |  |  | Inhibition | COWA test  (correct answers)  r = 0.705  p < 0.001 |
|  |  |  |  |  |  | Semantic Fluency test (correct answers)  r = 0.509  p = 0.013 |
|  |  |  |  |  | Cognitive flexibility | WCST  (Failure to Maintain Set)  T=-0.453  p = 0.013 |
|  |  |  |  |  |  | TMTA  (time to accomplish)  r = -0.416  p = 0.048 |
|  |  |  |  |  |  | TMTB  (time to accomplish)  r = -0.429  p = 0.041 |
|  |  |  |  |  |  | TMTB  (errors)  r = -0.662  p = 0.002 |
| Sayed et al.^60^ | ADHD-SC4 | ADHD = 72.5% | NA | - | NA | - |

| **Daily Functioning** | | | | | | |
| --- | --- | --- | --- | --- | --- | --- |
| Study | Assessment tool | Findings in SeLECTS patients | Comparison with HC | Statistics | Correlations with EFs | EFs assessment (statistics) |
| Miziara et al.^21^ | SPT, questionnaires | Poor school performance (patients report) | Statistically higher in SeLECTS | *p=0.008* | NA | - |
|  |  | Poor school performance (parents report) | Statistically higher in SeLECTS | *p=0.004* | NA | - |
|  |  | Poor school performance (teachers report) | Statistically higher in SeLECTS | *p=0.008* | NA | - |
| Lindgren et al.^30^ | Ansula Behaviour Rating Scale | Academic achievement, Attention and executive functions, social skills, questionnaire in total (patients report) | No significant difference | NA | NA | - |
|  |  | Academic achievement, Attention and executive functions, social skills, questionnaire in total (parents report) | No significant difference | NA | NA | - |
|  |  | Academic achievement, Attention and executive functions, social skills, questionnaire in total (teachers report) | No significant difference | NA | NA | - |
| Croona et al.^31^ | Parent- and teacher-based questionnaires | Distractibility (parents report) | Statistically higher in SeLECTS | *p<0.05* | NA | - |
|  |  | Concentration (parents report) | Statistically higher in SeLECTS | *p<0.01* | NA | - |
|  |  | Temper (parents report) | Statistically higher in SeLECTS | *p<0.001* | NA | - |
|  |  | Impulsiveness (parents report) | Statistically higher in SeLECTS | *p<0.05* | NA | - |
|  |  | Ability to understand instructions (parents report) | Statistically higher in SeLECTS | *p<0.05* | NA | - |
|  |  | Reading comprehension (teachers report) | Statistically higher in SeLECTS | *p<0.05* | NA | - |
| Zanaboni et al.^50^ | PedsQL | Total score | NA | - | BRIEF-P | I: -0.351 (p < 0.01)  S: -0.423 (p < 0.01)  EC: -0.325 (p < 0.01)  SM: -0.358 (p < 0.01)  In: -0.307 (p < 0.01)  WM: -0.310 (p < 0.01)  P/O: -0.394 (p < 0.01)  TM: -0.446 (p < 0.01)  OM: -0.281 (p < 0.01)  BRI: -0.335 (p < 0.01)  ERI: -0.379 (p < 0.01)  CRI: -0.354 (p < 0.01)  GEC: -0.388 (p < 0.01) |
|  |  | Physical health | NA | - | BRIEF-P | I: -0.261 (p < 0.01)  S: -0.333 (p < 0.01)  EC: -0.257 (p < 0.01)  SM: -0.279 (p < 0.01)  In: -0.276 (p < 0.01)  WM: -0.255 (p < 0.01)  P/O: -0.356 (p < 0.01)  TM: -0.290 (p < 0.01)  OM: -0.260 (p < 0.01)  BRI: -0.268 (p < 0.01)  ERI: -0.309 (p < 0.01)  CRI: -0.284 (p < 0.01)  GEC: -0.299 (p < 0.01) |
|  |  | Psychosocial health | NA | - | BRIEF-P | I: -0.339 (p < 0.01)  S: -0.403 (p < 0.01)  EC: -0.307 (p < 0.01)  SM: -0.344 (p < 0.01)  In: -0.274 (p < 0.01)  WM: -0.289 (p < 0.01)  P/O: -0.351 (p < 0.01)  TM: -0.459 (p < 0.01)  OM: -0.245 (p < 0.01)  BRI: -0.316 (p < 0.01)  ERI: -0.353 (p < 0.01)  CRI: -0.335 (p < 0.01)  GEC: -0.372 (p < 0.01) |
|  |  | Emotional functioning | NA | - | BRIEF-P | I: -0.251 (p < 0.01)  S: -0.312 (p < 0.01)  EC: -0.220 (p < 0.05)  SM: -0.197 (p < 0.05)  TM: -0.254 (p < 0.01)  BRI: -0.202 (p < 0.05)  ERI: -0.265 (p < 0.01)  GEC: -0.227 (p < 0.05) |
|  |  | Social functioning | NA | - | BRIEF-P | I: -0.199 (p < 0.05)  S: -0.294 (p < 0.01)  EC: -0.327 (p < 0.01)  SM: -0.327 (p < 0.01)  In: -0.298 (p < 0.01)  WM: -0.268 (p < 0.01)  P/O: -0.327 (p < 0.01)  TM: -0.358 (p < 0.01)  BRI: -0.231 (p < 0.05)  ERI: -0.299 (p < 0.01)  CRI: -0.278 (p < 0.01)  GEC: -0.333 (p < 0.01) |
|  |  | School functioning | NA | - | BRIEF-P | I: -0.377 (p < 0.01)  S: -0.384 (p < 0.01)  EC: -0.223 (p < 0.05)  SM: -0.344 (p < 0.01)  In: -0.265 (p < 0.01)  WM: -0.430 (p < 0.01)  P/O: -0.414 (p < 0.01)  TM: -0.537 (p < 0.01)  OM: -0.345 (p < 0.01)  BRI: -0.352 (p < 0.01)  ERI: -0.318 (p < 0.01)  CRI: -0.416 (p < 0.01)  GEC: -0.370 (p < 0.01) |

ADHD = attention deficit – hyperactivity disorder; ADHD-SC4 = attention deficit – hyperactivity disorder symptoms checklist 4; BRI = Behavioral Regulation Index; BRIEF-P = Behavior Rating Inventory of Executive Function - Parents’ Report; CBCL = Child Bheavioral Check- List; COWA = Controlled Oral Word Association Test; CRI = Cognitive Regulation Index; CRS = Conners’ Rating Scale; DCD = Developmental Coordination Disorder; DD = Developmental Dyslexia; DSM-IV-TR = Diagnostic and Statistical Manual of Mental Disorders, Fourth Edition, Text Revision; EC = emotional control; EFs = executive functions; ERI = Emotion Regulation Index; GEC = Global Executive Composite; HC = healthy controls; I = Inhibit; In = Initiate; K-SADS = Kiddie Schedule for Affective Disorders and Schizophrenia; MDD = Major Depressive Disorder; NA = not available; ODD = Oppositional Defiant Disorder; OM = Organization of Materials; P/O = Plan/Organize; PedsQL = Pediatric Quality of Life Inventory; S = shift; SAD = Seperation Anxiety Disorder; SeLECTS = Self Limited Epilepsy wit Centro-Temporal Spikes; SLD = Specific Learning Disorder; SM = self-monitor; SPT = School Performance Test; TM = Task-Monitor; TMTA = Trail Making Test, part A; TMTB = Trail Making Test, part B;WCST = Wisconsin Card Sorting Test; WM = Working Memory.
