# Supplementary material for: Executive functions and self‐limited epilepsy with centro‐temporal spikes: A scoping review
Source: Epileptic Disord. 2026 Jan 12;28(2):275–94. doi: 10.1002/epd2.70176 (PMC13084210; doi:10.1002/epd2.70176)
Supplement: Supplementary file 2 — Data S1. [file EPD2-28-275-s002.docx]

**Question 1:**

C. Both basic and high-order executive functions

Correct answer because: the review shows that children with SeLECTS often display difficulties across a wide range of executive functions, from basic (e.g., attention, working memory) to higher-order (e.g., planning, flexibility) EFs.

Why the others are wrong:

A. Only emotional regulation: emotional regulation is not the main or only domain affected.

B. Only basic executive functions: high-order functions are also reported as impaired.

D. Only attention and memory: these are affected but not exclusively.

E. Only language-related functions: the review emphasizes executive function deficits, not just language skills.

**Editor comments:**

D. Implement tailored neuropsychological monitoring for at-risk children

Correct because: the review recommends individualized cognitive monitoring to detect and manage executive function difficulties.

Why the others are wrong:

A. Avoid all use of ASM: ASMs are sometimes necessary.

B. Delay treatment until cognitive decline: There can be other clinical reasons to start treatment.

C. Standardized education plans for all: not all children need the same plan.

E. Refer all for surgery: surgery is not indicated in SeLECTS.

**Question 3:**

**Editor Comments:**

Correct answer: B. They may lack sensitivity to subtle EFs deficits

Correct because: standard tests often miss mild impairments in executive functions.

Why the others are wrong:

A. They are not administered frequently enough: patients with SeLECTS are usually not tested if school performance is satisfying

C. Too difficult for children with epilepsy: tasks are adapted for age and cognitive skills.

D. Focus only on behavioral problems: assessments target cognition, not only behavior.

E. Influenced by socioeconomic background: this can be a possible confounding factor, but not the key reason.
